# Severity scoring system of COVID-19 in Egyptian medical personnel versus non-medical personnel: a prospective cohort study

**DOI:** 10.1186/s43055-022-00774-4

**Published:** 2022-05-16

**Authors:** Alaa Mohamed Reda, Lina Tarek Hablas, Rania Sobhy Abou Khadrah

**Affiliations:** Faculty of Medicine, Tanta University, El-Geish Street, Tanta, Gharbia Governorate Egypt

**Keywords:** COVID-19, Medical personnel, CT (computed tomography)

## Abstract

**Background:**

Few studies tried to detect the risk of developing COVID-19 (corona virus disease 2019) between different groups of workers. Health care workers are more likely to have severe form of COVID infection. The objective of our study is to compare the severity scoring system of COVID infection between medical and non-medical personnel by CT (computed tomography). This study started from 1 May, 2020, to 30 December, 2021. CT study of the chest for 1200 infected patients with COVID-19 (600 were medical stuff and 600 persons of non-medical staff) was done in five health quarantine centres in Egypt. CT findings were reviewed in relation to the severity of symptoms. The results of the two groups were compared to each other.

**Results:**

CT findings were more frequent and more severe in the medical staff group, including that the number of consolidative patches mixed with areas of ground glass attenuation in medical staff group was (37.2%) versus non-medical staff group was (22.2%), with p-value (*0.001), diffuse lobar involvement was in (150 severe cases) in medical staff group versus in 67 severe cases of non-medical staff group and had significant difference (*p *value *0.005), fibrosis (*p *value *0.002) and more opportunity to get severe form of infection increased in medical personnel rather than control group (*p* value *0.001) which may be due to limited health care facilities in protection against infection especially in developing countries and more contact during work time with infected persons and high viral load.

**Conclusion:**

The current study results show that severity score is higher in the medical personnel than non-medial personnel.

## Background

The World Health Organization (WHO) declared the novel coronavirus disease, COVID-19; a public health emergency of international concern [1], and by 11 March 2020, the COVID-19 outbreak was declared a global pandemic [2, 3]. According to Coronavirus disease 2019 (COVID-19) situation report from WHO, totally 191,127 cases of patients were laboratory confirmed and amongst them 7807 patients died by 18 March 2020 [4, 5]. Radiological evaluation of patients with SARS-COVID 19 infection particularly by chest computed tomography (CT) has a reported high sensitivity and enhances the clinical decision that is based on the degree of lung affection [6, 7]. Yang and his colleagues [8] introduce a severity scoring system (CT-SS) that depends on the degree of lung affection in chest CT and is recommended to be used for quick assessment of pulmonary affection. Moreover, in March 2020, the Dutch Radiological Society developed another scoring system based on chest CT and patient’s data; the COVID-19 Reporting and Data System (CO-RADS) included data of clinical finding and laboratory test results in addition to CT records. The degree of suspicion ranged from very low to very high (CO-RADS categories 1–5), while category 0 reflects negative infection and category 6 establishes RT-PCR-positive COVID infection at time of examination [9].

Few studies had focused on the difference in the risk of developing severe form of COVID-19 infection especially among the medical compared to non-medical individuals [10].

Omicron, a new variant of the coronavirus, has put the world on red alert. Reports emerged from South Africa on November 24, and 2 days later the World Health Organization dubbed Omicron a variant of concern. The news rattled financial markets and prompted countries to close their borders, though authorities found within a week that the variant was already in Australia, China, Europe, and the USA [11].

The current study tried to assess the medical personnel’ COVID infection and detect if there are any predisposing factors in developing severe form of infection for early and proper management; to identify if the medical workers had more liability for developing severe form of infection due to many factors as higher virus load and exposure to more aggressive viral serotypes. The main objective of our study is a prospective comparing medical and non-medical staff by severity score in five health care quarantine centres in Egypt, aiming for more self-protection, more caring and early diagnosis of COVID by CT.

## Patients and methods

### Study design, setting and ethical considerations

This study was a prospective cohort study. The study participants (1200 patients) were hospitalized in five quarantine centres in Egyptian health care system with positively proven PCR laboratory testing of respiratory secretions obtained by nasopharyngeal swabs, 600 of them were of medical staff (doctors, nurses and radiographers), another 600 cases of non-medical staff, all of them underwent non-contrast CT study of the chest. In addition to sociodemographic data (that included age, sex, occupation, body mass index (BMI), concomitant/previous diseases and smoking habit), exposure history, clinical manifestations were collected including severity and duration course of symptoms. This study started from 1 May, 2020, to 30 December, 2021.

### Inclusion criteria

The study included all patients above 18 years who were admitted to quarantine centre in the Egyptian health care system with positive PCR testing for COVID-19.

### Exclusion criteria

Pregnant women and children under 18 years old were excluded from this study due to hazards of radiation from CT, and patients with chronic lung diseases’ such as tuberculosis, interstitial lung diseases and pulmonary malignancy to avoid interference with radiological presentation of COVID-19.

**Fig. 1:**
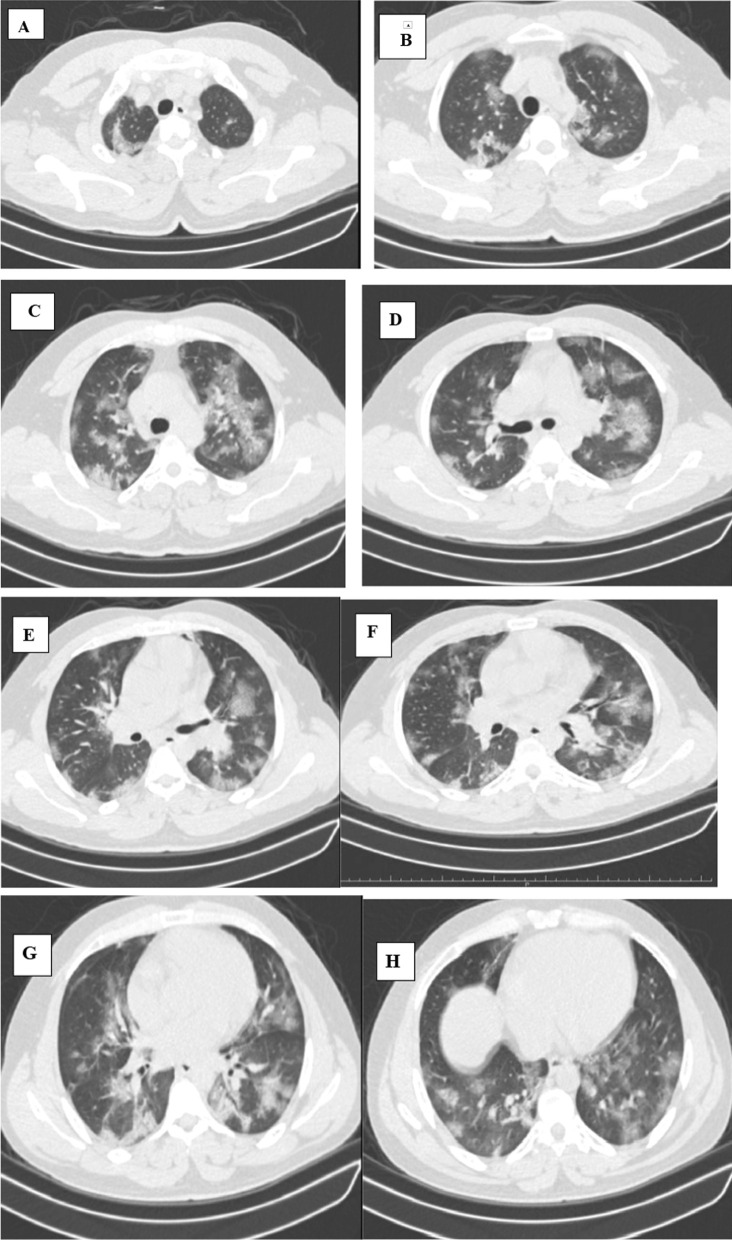
A 45-year-old infected patient from non-medical group with COVID-19 presented with dyspnea and fever. **A**, **B** Axial chest CT cuts (lung window) showing multiple bilateral sub-pleural patchy areas of ground glass attenuation and sub-segmental consolidations in both apical segments of both upper lobes (0–25%). **C**–**F** Axial chest CT cuts (lung window) showing involvement of anterior segments of both upper lobes and apical segments of both lower lobes (25–50%). **G**, **H** Axial chest CT cuts (lung window) showing involvement of lingula, middle lobe and both lower lobes (30%) by patchy sub-segmental consolidative areas, curvilinear band and ground glass pattern. CT severity score (CTSS) is 9/25

**Fig. 2:**
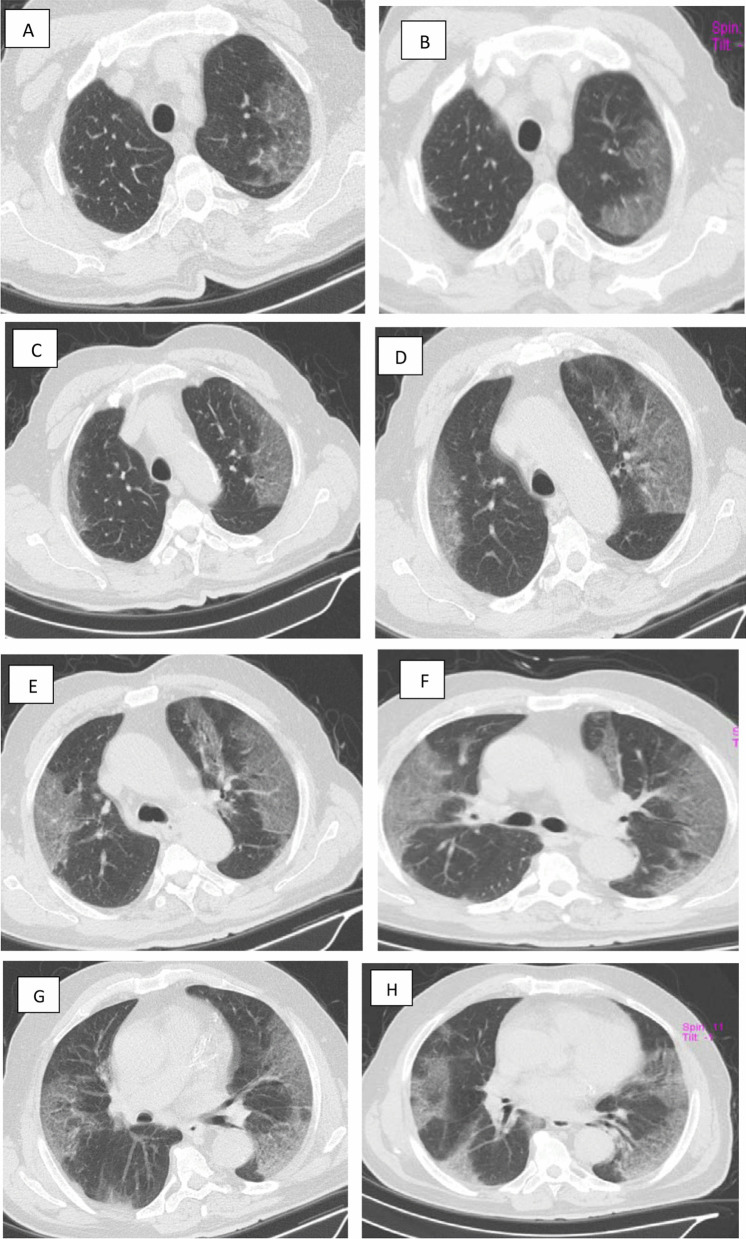

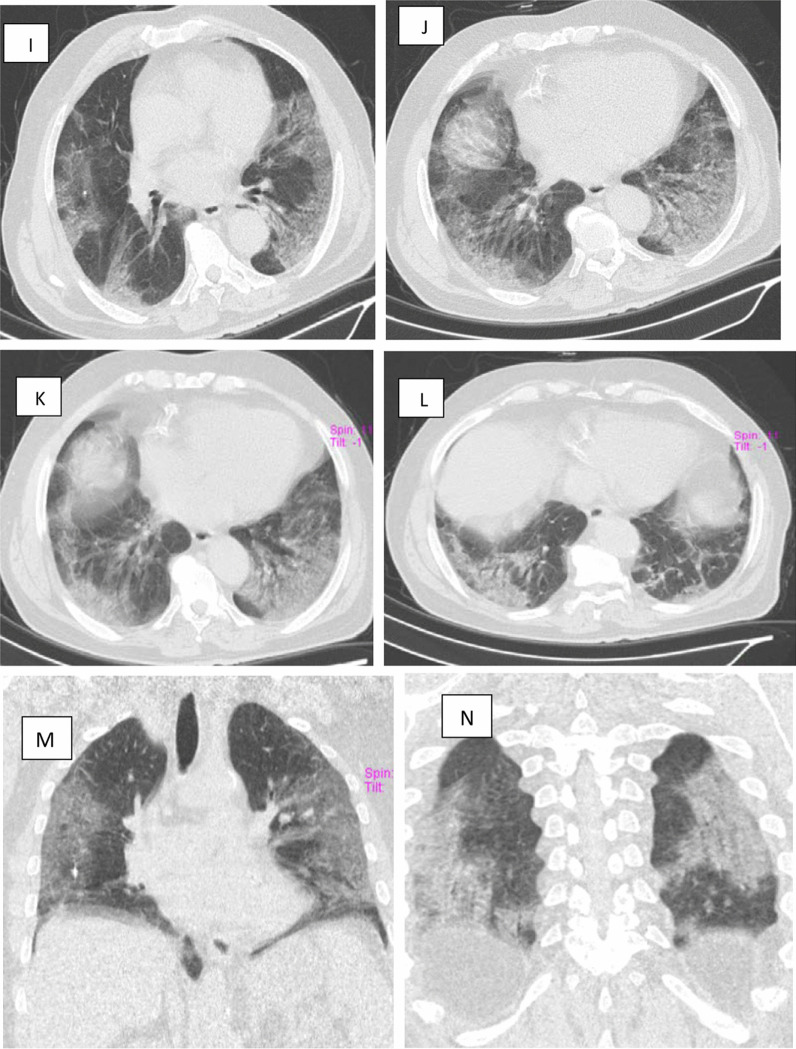
A 61-year-old infected nurse with COVID-19 presented with dyspnea, cough and decreased O2 saturation level to 83 percent. **A**–**C** Axial chest CT cuts (lung window) showing multiple bilateral sub-pleural patchy areas of ground glass attenuation and sub-segmental consolidations in both apical segments of right and left upper lobes (5% and 35%, respectively). **C**–**H** Axial chest CT cuts (lung window) showing involvement of anterior segments of both upper lobes and apical segments of both lower lobes (25–50%). **I**–**L** Axial chest CT cuts (lung window) showing involvement of lingula, middle lobe and both right and left lower lobes (10–25% 35 & 75%, respectively) by patchy sub-segmental consolidative areas, curvilinear band and ground glass pattern.** M** and** N**. coronal cuts showing subpleural ground glass areas with sub-segmental sub-pleural consolidative patches. CT severity score (CTSS) is 14/25

**Fig. 3:**
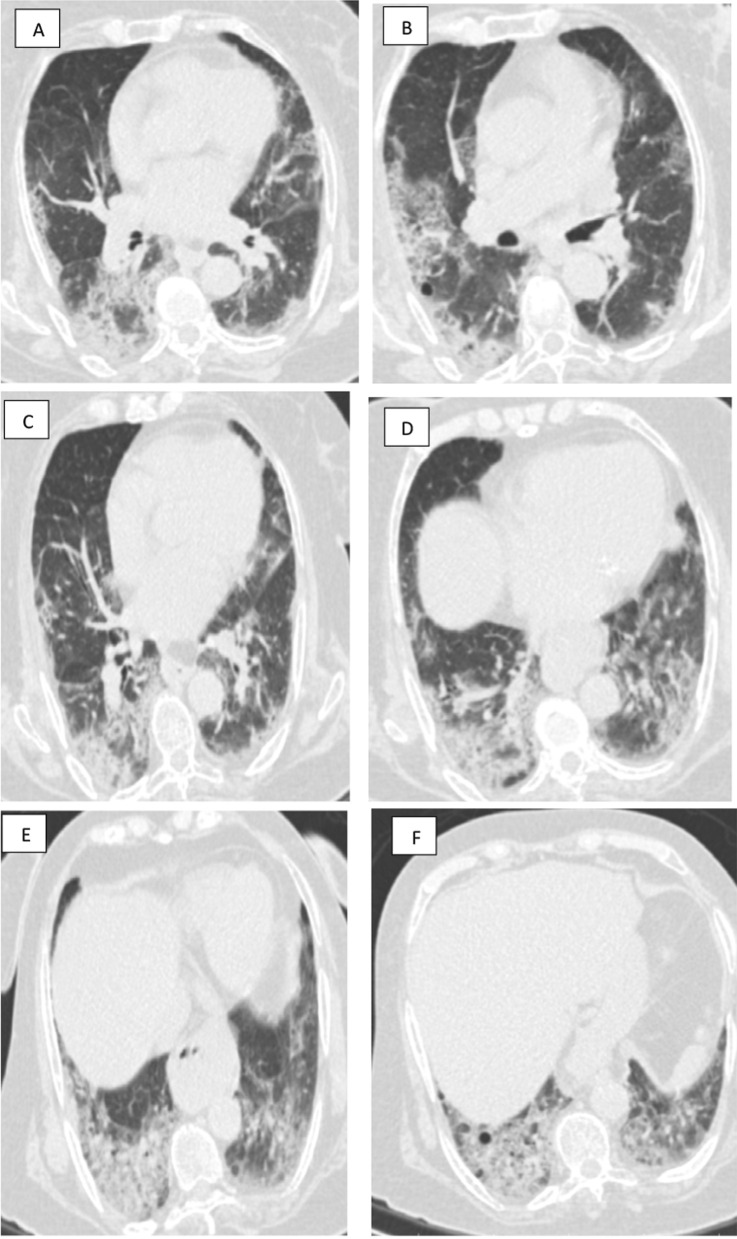
A 52-year-old infected doctor with COVID-19 presented with dyspnea and on low flow oxygen support. **A**, **B** Axial chest CT cuts (lung window) showing multiple bilateral sub-pleural patchy areas of ground glass attenuation and sub-segmental consolidations in both upper lobes (20%). **C**, **D** Axial chest CT cuts (lung window) showing involvement of middle lobe and lingula (5–10%). **E**, **F** Axial chest CT cuts (lung window) showing involvement of right and left lower lobes (80% and 75%%) by patchy sub-segmental consolidative areas, curvilinear band and ground glass pattern, interstitial thickening, reticulo-nodular infiltrates and fibrosis, mild bronchial and vascular dilatation. CT severity score (CTSS) is 16/25

PCR assay was performed by nasopharyngeal swab. Clinical and laboratory assessment consisted of body temperature, PaO2, and C-reactive protein. In addition, the present study differentiated between low-flow oxygenation (nasal cannula, face mask), high-flow oxygenation (Venturi mask, CPAP) and mechanical ventilation through an endotracheal tube. The time interval from CT performance and oxygenation support was estimated. In-hospital deaths and healed patients’ discharge dates were also detected. The clinical features of confusion (mental test score of 8 or less), urea, respiratory rate and blood pressure were also acquired for the risk of mortality (Figs. 1, 2, 3).

### CT technique and image interpretation

No specific preparation was done only all metallic items were removed as bra, coins, etc. High-resolution non-contrast CT study of the chest was performed for all patients using 320 multi-slice CT scanner, Aquilion one, TOSHIBA medical systems, GE lightspeed VCT-64 Multi-slice, GE Bright speed Elite-16 Multi-slice, GE optima CT 660 (64 Multi-slice) and Toshiba prime 160 Multi-slice.

Radiation exposure was adapted according to body habitus of the patients. All scans were obtained with the patient in the supine position during end-inspiration (breath hold) without intravenous contrast material. Field of view included the whole chest. CT was done using automatic tube current modulation (ranging from 100 to 380 mA). Other CT parameters are mentioned in the following table:

**Table:** 

	Tube voltage (KVp)	Pitch	Slice thickness (mm)
320 multi-slice CT scanner, aquilion one, TOSHIBA medical systems	100	1.1	1
GE lightspeed VCT-64 multi-slice	120	1.5	1–2
GE bright speed elite-16 multi-slice	120	1	1
GE optima CT 660 (64 multi-slice)	120	0.76–1.22	1
Toshiba prime 160 multi-slice	100	1.1	1

The dataset was anonymised and exported to a dedicated segmentation suite for image computing equipped with a semiautomated segmentation algorithm (chest imaging platform). Using a viewing console, images were examined in axial cuts and reconstructed by multi planner reformatting into sagittal and coronal views. After each examination, surface disinfection was performed with ethanol 60–70% and 0.1% sodium hypochlorite and passive air exchange for 45–90 min.

CT images were reviewed by two experienced senior consultants with approximately 10 years of experience each, and final opinions were reached by consensus. For disagreement between the two primary radiologists' interpretations, a third fellowship-trained cardiothoracic radiologist with 8 years of experience adjudicated a final decision (whose subspecialty where chest imaging).

For each of the 1200 patients, CT images were reviewed and evaluated for the presence of unifocal or multifocal ground-glass attenuation (which is defined as hazy un sharply demarcated area of increased pulmonary attenuation with preserved bronchial and vascular margins), peripherally located (sub-pleural) patchy areas of consolidation, number of lobes affected, bronchial or vascular dilatation, sub pleural bands, reversed halo sign, presence of nodules, pleural effusion and mediastinal lymphadenopathy (larger than 1 cm in short axis dimension).

### Chest CT severity score

Chest CT score is calculated per each of the 5 lobes based on the extent of parenchymal involvement, as follows: (0) no involvement; (1) < 5% involvement; (2) 5–25% involvement; (3) 26–50% involvement; (4) 51–75% involvement; and (5) > 75% involvement. The resulting total CT score is the sum of each individual lobar score and ranges from 0 to 25 (mild: 1–10, moderate 11–20 and severe score 21–25).

### Statistical analysis

All data were collected and analysed by number and percentage. Pearson Chi-squared test was done for nominal variables. Significant values cut-off was set at *p* ≤ 0.05. Calculations were performed on a standard PC unit using a statistical analysis program (Minitab, version15).

## Results

A total of 1200 infected cases with positive PCR for COVID19 were included (600 of them were medical staff and 600 persons of non- medical staff).

Regarding the age, the more frequent age group was from 50 to  < 60 years old representing 32.5% out of the total 1200 studied cases, while regarding the gender; males were more frequent in both medical and non-medical groups presented by 57% and 68.5%, respectively, as seen in Table 1 that also shows the analysis of sociodemographic data between medical personnel (hospital acquired infection) and non-medical infected cases (community acquired infection) of different variables; no significant difference between both groups regarding age, sex, risk factors, apart from smoking with *P* value (*0.004), as well as significant difference regarding to laboratory investigations (CRP, D-dimer and interleukin-6) with *P* = values = *0.004, *0.35 and *0.001).

**Table 1:** Comparison of sociodemographic characteristics of the studied patients (*N* = 1200)

		Groups							Chi-square test
		Medical personnel *N* = 600			Non-medical personnel *N* = 600		Total number *N* = 1200		
		*N*	%		*N*	%	*N*	%	*P* value
Age groups (years)	18 to < 30	22	3.6%		9	1.5%	31	2.5%	0.9
	30 to < 40	117	19.5%		81	13.5%	198	16.5%	0.79
	40 to < 50	143	23.8%		127	21.1%	270	22.5%	0.57
	50 to < 60	181	30.1%		210	35%	391	32.5%	0.68
	60 to < 70	70	11.6%		85	14.1%	155	12.9%	0.83
	70 to < 80	67	11.1%		88	14.6%	155	12.9%	0.9
Sex	Male	342	57%		411	68.5%	753	62.75%	0.88
	Female	258	43%		189	31.5%	447	37.25%	0.7
Smoking	Yes	217	36%		497	82.%	714	59.5%	*0.004
	No	384	64%		103	18%	487	40.5%	
Occupation	Doctors	319	53.1%	Carpenters	43	7.1%			
	Nurses	110	18.3%	Teachers	269	44.8%			
	Radiographers	94	15.6%	Farmers	104	17.3%			
	Assistant staff	77	12.8%	Drivers	184	30.6%			
Risk factors	Diabetes mellitus	33	5.5%		170	28.33%	203	16.9%	0.81
	Bronchial asthma	197	32.83%		186	31%	383	31.9%	1
	Auto-immune disease	9	1.5%		44	7.3%	53	4.4%	0.77
	Obesity (BMI more than 30)	105	17.5%		141	23.5%	246	20.5%	0.84
	No risk factors	256	42.66%		59	9.8%	315	26.2%	*0.04

N, number

*Significant at *p* < 0.05

Regarding the symptomatology, the median body temperature was 38.5 °C and 39.1 °C in medical and non-medical personnel groups, respectively, non-productive cough and non-exertional dyspnoea is more frequent and severe in the first group, with significant difference between both groups (*P* value = 0.05* and 0.003*, respectively), as in Table 2.

**Table 2:** Comparison between medical personnel and non-medical personnel group as clinical manifestation (*N* = 1200)

Symptom	Medical personnel group (*N* = 600)						Non-medical personnel group (*N* = 600)						
	Mild *N* = 120 (20%)		Moderate *N* = 233 (38.8%)		Severe *N* = 247 (41.66%)		Mild *N* = 358 (59.66%)		Moderate *N* = 152 (25.33%)		Severe *N* = 90 (15%)		*P* value
	*N*	%	*N*	%	*N*	%	*N*	%	*N*	%	*N*	%	
Fever	34	28.3%	55	23.6%	21	8.5%	50	13.9%	36	23%	8	8.8%	0.99
Fatigue	10	8.3%	18	7.72%	5	2%	17	4.7%	10	6.5%	2	2.2%	0.82
Cough	5	4.1%	30	12.8%	66	26.7%	21	5.8%	22	14.4%	20	22.2%	*0.05
Dyspnea	2	1.6%	39	16.7%	78	31.5%	61	17%	39	25.6%	22	24.4%	*0.003
Diarrhoea	4	3.3%	11	4.7%	5	2%	22	6.1%	15	9.8%	2	2.2%	0.97
Headache	10	8.3%	28	12%	6	2.4%	101	28%	3	1.9%	1	1.1%	0.66
Loss of smell and taste	53	44.1%	31	13.3%	3	1.2%	81	22.6%	12	7.8%	1	1.1%	0.59
Confusion	0	0	4	1.7%	26	10.5%	2	0.55%	7	4.6%	21	23.3%	*0.45
Chest pain	2	1.6%	17	7.2%	37	14.9%	3	0.83%	8	5.2%	13	14.4%	0.98

N, number

*Significant at *p* < 0.05

In the medical stuff group, the median PaO2 was 69 mmHg (IQR, 44–95). Out of the 600 cases of medical personnel’s; 68.3% of them required oxygenation support, as follows: 18% required low-flow oxygenation (nasal cannula and facial mask); 22% needed high-flow oxygenation (helmet CPAP); and 28.3% (170 cases) underwent mechanical ventilation with an endotracheal tube. However, in the non-medical stuff group, the median PaO2 was 80 mmHg (IQR, 60–100), 55% of them required oxygenation support, as follows: 30% required low-flow oxygenation (nasal cannula and facial mask), 17% was on high-flow oxygenation (helmet CPAP) and 8% (50 cases) underwent mechanical ventilation with an endotracheal tube (Table 3).

**Table 3:** Comparison between medical personnel and non-medical personnel groups as regards laboratory findings (*N* = 1200) (range, mean ± SD):

Laboratory findings	Medical personnel group (*N* = 600)						Non-medical personnel group (*N* = 600)						*P*
	Mild		Moderate		Severe		Mild		Moderate		Severe		
WBCs (10^3^/μL)	8.5–11.7	10.6 ± 4	6.1–9.3	8.9 ± 3	4.6–6	5.4 ± 2	7.5–10	10.9 ± 4	6.1–9.3	9.8	5–7.1	6.4 ± 3	0.78
Lymphocytes (10^3^/μL)	20–40	22.9 ± 7	13–19	15.9 ± 5	7–9	8.1 ± 3	23–43	25.9 ± 2	14–18.8	15 ± 1	8–9.2	8 ± 2	0.65
CRP (mg/L)	5–7.5	5 ± 1	10–30	15 ± 4	64–305	109 ± 6	3.6–5.5	4.8 ± 1	8.9–21	11 ± 3	40–190	86 ± 9	*0.004
D dimer (μg FEU/ml)	0.01–0.3	0.07 ± 0.02	0.4–0.6	0.5 ± 0.03	0.9–2.1	1.5 ± 0.07	0.01–0.1	0.04 ± 0.02	0.4–0.6	0.5 ± 0.01	0.7–1.8	1.4 ± 0.05	*0.35
Ferritin (ng/ml)	25–270	76 ± 39	143–398	176 ± 48	937–2803	1676 ± 77	21–198	61 ± 29	127–284	154 ± 40	860–1701	992 ± 87	0.9
Interleukin-6 (pg/ml)	0.5–0.9	0.7 ± 0.01	1.2–3.9	1.6 ± 0.9	9.5–12.2	10.7 ± 2	0.2–1.2	0.6 ± 1	6.5–10	7 ± 2	7.8–11	8.2 ± 2.1	*0.001

Independent t test

Chi-square test

*P considered significant if *P* < 0.05. Continuous data represented as mean and standard deviation (SD), and categorical data as number and percentage (%)

Regarding the lung abnormalities of both medical and non-medical groups, fibrosis, emphysematous changes show significant difference between both groups (*P* value = 0.02* and 0.01*), respectively.

Regarding the laterally of lung affection, no statistically significant difference between both lung fields affection between the two groups (*P *value = 0.8, 0.6 and 0.99) for right, left and both sides, respectively.

Moreover, the lobar involvement in severe cases of both groups was significant in involving right lower and left lower lung lobes (*P* value = *0.001 and *0.002, respectively), with more lobar number involvement (4lobes and 5 lobes) in medical than non-medical groups, which is statistically significant difference (*P* value = 0.003*, 0.002*) (Tables 4, 5, 6, 7, 8).

**Table 4:** Comparison between medical personnel and non-medical personnel groups, as regards lung abnormality (*N* = 1200)

	Medical personnel group (*N* = 600)						Non-medical personnel group (*N* = 600)						*P* value
	Mild (*N* = 120)		Moderate (*N* = 233)		Severe (*N* = 247)		Mild (*N* = 358)		Moderate (*N* = 152)		Severe (*N* = 90)		
	*N*	%	*N*	%	*N*	%	*N*	%	*N*	%	*N*	%	
^a^Lung abnormality	62	51.66%	59	25.3%	70	28.3%	184	51.3%	55	36.1%	13	14.4%	0.7
Only consolidation													
Ground-glass opacities mixed with consolidation	30	25%	76	32.6%	92	37.2%	143	39.9%	66	43.4%	20	22.2%	*0.001
Pleural effusion	20	16.6%	29	12.4%	22	8.9%	18	5%	19	12.5%	15	16.6%	0.56
Emphysema	5	4.1%	34	14.5%	30	12.1%	13	3.6%	12	7.8%	23	25.5%	*0.001
Fibrosis	3	2.5%	35	15%	33	13.3%	0	0	0	0	19	21.1%	*0.002

N, number; NS, non-significant

*Significant at *p* < 0.05

**Table 5:** Comparison between medical personnel and non-medical personnel groups, as regards laterality of lung involvement (*N* = 1200)

Laterality	Medical personnel group (*N* = 600)						Non-medical personnel group (*N* = 600)						*P* value
	Mild		Moderate		Severe		Mild		Moderate		Severe		
	*N*	%	*N*	%	*N*	%	*N*	%	*N*	%	*N*	%	
Right side	48	40%	76	32.6%	35	14.17%	102	28.4%	32	21%	18	20%	0.8
Left side	54	45%	59	25.3%	49	19.8%	204	56.9%	63	41.4%	12	13.3%	0.6
Bilateral	18	15%	98	42%	163	65.9%	52	14.5%	57	37.5%	60	66.6%	0.99

**Table 6:** Comparison between medical personnel and non-medical personnel groups, as regards distribution of lobar lung involvement (*N* = 1200)

Lobar involvement	Medical personnel group (*N* = 600)						Non-medical personnel group (*N* = 600)						*P* value
	Mild		Moderate		Severe		Mild		Moderate		Severe		
	*N*	%	*N*	%	*N*	%	*N*	%	*N*	%	*N*	%	
Rt upper lobe	11	9.1%	28	12%	10	4%	26	7.2%	11	7.2%	4	4.4%	0.7 NS
Rt middle lobe	9	7.5%	8	3.4%	2	0.8%	9	2.5%	2	1.3%	4	4.4%	0.6 NS
Rt lower lobe	28	23.3%	40	33.3%	23	9.3%	76	21.2%	19	12.5%	10	11.1%	*0.001
Lt upper lobe	24	20%	25	10.7%	15	6%	99	27.6%	24	15.7%	3	3.3%	0.9 NS
Lt lower lobe	30	25%	34	14.5%	34	13.7%	105	29.3%	33	21.7%	9	10%	*0.002

**Table 7:** Comparison between medical personnel and non-medical personnel groups, as regards number of lobes involved (*N* = 1200)

Number of lobes involved	Medical personnel group (*N* = 600)						Non-medical personnel group (*N* = 600)						*P* value
	Mild		Moderate		Severe		Mild		Moderate		Severe		
	*N*	%	*N*	%	*N*	%	*N*	%	*N*	%	*N*	%	
1 lobe	67	55.8%	12	5.1%	2	0.8%	214	59.7%	11	7.2%	9	10%	0.06 NS
2 lobes	22	18.3%	50	21.4%	17	6.88%	63	17.5%	33	21.7%	11	12.2%	0.9 NS
3 lobes	16	13.3%	62	26.6%	19	7.6%	60	16.7%	17	11.1%	12	13.3%	0.06 NS
4 lobes	15	12.5%	60	25.7%	50	20.2%	21	5.8%	41	26.9%	15	16.6%	*0.003
5 lobes	0	0	49	40.8%	159	64.3%	0	0	50	32.8%	43	47.7%	*0.002

*p value < 0.05, **p value < 0.01

**Table 8:** Comparison between medical personnel and non-medical personnel groups, regarding distribution of lung involvement (*N* = 1200)

Distribution	Medical personnel group (*N* = 600)						Non-medical personnel group (*N* = 600)						*P* value
	Mild		Moderate		Severe		Mild		Moderate		Severe		
	*N*	%	*N*	%	*N*	%	*N*	%	*N*	%	*N*	%	
Central only	23	19.1%	33	14.1%	30	12.1%	19	5.3%	15	9.8%	11	12.2%	0.07 NS
Peripheral only	85	70.8%	114	48.9%	67	27.1%	312	87.1%	70	46%	12	13.3%	0.09 NS
Diffuse	12	10%	86	36.9%	150	60.7%	27	7.5%	67	44%	67	74.4%	*0.005

*p value < 0.05, **p value < 0.01

The distribution of lung abnormalities was significant difference in diffuse affection between both groups, with *P* value = 0.005*.

Regarding the outcome of the studied cases, there was no significant difference of the hospital stay time between both groups; however, the majority (99.5%) of the severe cases of medical group need to admit in ICU, with 150 (60.7%) of them needed to mechanical ventilator, with significant difference, *P* value = 0.003*, 0.02*, respectively, as seen in Table 9.

Table 9 shows that the number of deaths were higher in medical group rather than the non-medical group, out of 247 severe cases of the medical group; 179 (72.4%) died, while out of 90 severe cases of the second group, 37 (41.1%) died, with statistically significant difference of the number of deaths between the two groups, *P * value = 0.03*.

**Table 9:** Prognosis and outcome of COVID-19 cases in both medical personnel and non-medical personnel (*N* = 1200)

	Medical personnel group (*N* = 600)			Non-medical personnel group (*N* = 600)			*P*
	Mild (*N* = 120)	Moderate (*N* = 233)	Severe (*N* = 247)	Mild (*N* = 358)	Moderate (*N* = 152)	Severe (*N* = 90)	
Need ICU	3 (2.5%)	77 (33%)	246 (99.5%)	0	34 (22.3%)	88 (97.7%)	*0.003
Need MV	0	20 (8.5%)	150 (60.7%)	0	9 (5.9%)	41 (45.5%)	*0.02
ARDs	5 (4.1%)	13 (5.5%)	81 (32.7%)	2 (0.5%)	9 (5.9%)	22 (24.4%)	*0.004
Deaths	2 (1.6%)	10 (4.2%)	179 (72.4%)	0	4 (2.6%)	37 (41.1%)	*0.03
LOS (day)	7 ± 3	12 ± 5	30 ± 12	5 ± 2	10 ± 4	22 ± 10	0.9

ICU, intensive case unit; MV, Mechanical ventilation; ARDs, adult respiratory distress syndrome; LOS, length of stay

Independent t test

Chi-square test

*P considered significant if *P* < 0.05. Continues data represented as mean and standard deviation (SD), and categorical data as number and percentage

## Discussion

Since the beginning of first epidemic of COVID-19 with first officially reported case (COVID-19) in last December, 2019 in Wuhan, China, the outbreak has evolved and continuous to second and recently third wave of pandemic into a global public health crisis with an extensive number of infected patients, causing devastating deaths all over the world [4]. So far, the COVID-19 pandemic is still ongoing, and the vaccination is seemed to be the only hope to stop this pandemic specially in high-risk group as doctor, nurses, and their assistants.

This study tried to compare the severity of COVID-19 infection between non-medical infected group and medical Egyptian infected persons in five centres, confirmed to be COVID-19 by laboratory tests aiming to guide if the medical personnel are in urgent to be vaccinated or not. Typical CT appearance of COVID-19 Was described firstly by Chung et al. [5] and then has been confirmed by several reports [6]. Depended on these findings, Liu et al. have proposed a CT scoring grade for severity of lung damage based on a “total severity score” [4]**.**

This cohort study revealed that male more common to get infection *n* = 753 (62.75%) in contrast to study of Zaina Al Maskaria et al. [12]^.^ which stated that 64% of infected medical personnel were female and matched with result of Sabetian et al. [13]. The cause of this predominance remains unknown, but it may be due to more likely to long time of exposure to infected patients or co-associated other medical condition like diabetes or chronic lung disease.

Egyptian medical personnel had severe form of infection when compared to other non-medical group and this risk increased in doctors (53.1%), followed by nurses (18.3%) and then radiographers (15.6%) and assistance staff (12.8%); these results were not matched with result of Sabetian et al. [13] and Zaina Al-Maskari et al. [12]

It was observed that the number of consolidative patches mixed with areas of ground glass attenuation in medical staff group was (37.2%) versus non-medical staff group was (22.2%), with *p* value (*0.001), diffuse lobar involvement was in (150 severe cases) in medical staff group versus in 67 severe cases of non-medical staff group and had (*p* value *0.005), fibrosis (*p* value *0.002) and so, more opportunity to get severe form of infection is higher and more frequent in medical personnel (*p* value *0.001) which may be due to limited health care facilities in protection against infection especially in developing countries and more contact during work time with infected persons; these results were in tune with results of Zaina Al Maskaria et al. [12] who explained that but more liability to get infection from colleague during time break without masks and strict infection control procedures.

Out of 600 medial personals, 22% needed high-flow oxygenation (helmet CPAP); and 28.3% (170 cases) underwent mechanical ventilation with an endotracheal tube, while in non-medical control group 17% was on high-flow oxygenation (helmet CPAP) and 8% (50 cases) underwent mechanical ventilation with an endotracheal tube and this matched with result of Chu et al. [14] who reported that the severity of the disease was more pronounced and more likely to need mechanical ventilation in medical staff group.

## Conclusions

Finally to conclude that the CT severity of COVID-19 infection, more lobar number involvement, more number of ICU admission, mechanical ventilation and number deaths are higher in medical personnel rather than non-medical group. Further studies to include all Egyptian quarantine centres and more countries are recommended.

### Limitations

The current study was limited for a period of time with relative underestimation of number of infected medical staff due to underreport of many undiagnosed cases, and not all quarantine centres in Egypt were included in our study, also underestimation of the symptoms during recording data as the infected medical personnel might progress to more severe symptoms and no recorded different patient profiles and treatment protocols between different centres had resulted in discrepancy in COVID-19 pneumonia severity and in the hospital outcome. Lastly asymptomatic cases were not included.

Our recommendations are for more future research to include a larger sample size over longer periods of time and multi-centric all over the world for comparison between different viral strains worldwide.

## Data Availability

The datasets used and/or analysed during the current study are available from the corresponding author on reasonable request.

## Abbreviations

CT: Computed tomography
COVID-19: Corona Virus Disease 2019
SARS: Severe acute respiratory syndrome
CORAD: Covid-19 Reporting and Data System
PCR: Polymerase chain reaction
DM: Diabetes mellitus
BMI: Basal Metabolic Index (45%)
